# 

**Published:** 1895-06

**Authors:** 


MESSRS. William R. Warner & Co., of Philadelphia, have received from the Belgian government a decoration which is an additional tribute for the excellence and superiority of the firm's ready-coated pills and other pharmaceutical products, for which the house has a great name. The decoration is of most beautiful design, in gold and white enamel, taking the form of a Maltese cross, on the center of which, on a blue ground, is the inscription. A wreath in blue and gold surmounts the cross, the whole being topped by ribbon, tied in a bow, of the national colors.—*Philadelphia Inquirer.*

THERE comes to us from the *Ladies' Home Journal*, a very artistically gotten-up illustrated booklet of over 250 pages, called "5,000 Books," which serves as an easy guide to the best books in any department of reading. This guide is very well done. The best literary experts of New York, Boston and Philadelphia were engaged by the *Journal* to select the 5,000 books which it presents as the most desirable for a home library, and their work has been admirably carried out. Very clear, explanatory comments are given by these men of books, and besides there are given not less than 160 portraits of leading authors. No book will, perhaps, do so much to extend good reading as this guide, so carefully gotten up, so beautifully printed, and so generously offered, free of any charge, by the publishers of the *Ladies' Home Journal.* "5,000 Books" is unquestionably the best and easiest guide to a wise selection of books that has been issued for a long time.

---

ANOTHER BOOK ON KOLA.—That the old, and yet new drug, Kola, is becoming of interest here as well as abroad, is apparent from the fact that Johnson & Johnson have issued three large editions of their monograph, Kola Illustrated. The last one, while small in size, is by far the handsomest of any in make-up and illustrations. It is brimful of concise and clear statements regarding the history, chemical properties, physiological action and therapeutic uses of the drug, and contains some very interesting reports upon the use of the drug in physical and mental labor, athletic training and exercise.

The author states that it has been written for "the busy physician." Certainly it contains all that is old and much that is entirely new, and in such a way as to be available to any who desire to study this drug.

Physicians may secure a copy by addressing the publishers, Johnson & Johnson.

---

THE *Medical Chronicle*, conducted by an editorial committee consisting of the professors and lecturers of the medical department of the Owens College and published at Manchester, is one of the very best of the English medical magazines. It is edited by Professors D. J. Lesch, William Jap Sinclair and A. H. Young. A new volume began with the April number, 1895, which presents every evidence of a progressive improvement that keeps it abreast of the medical science of the period.

